# Retraction Note: A83-01 inhibits TGF-β-induced upregulation of Wnt3 and epithelial to mesenchymal transition in HER2-overexpressing breast cancer cells

**DOI:** 10.1007/s10549-024-07371-1

**Published:** 2024-05-10

**Authors:** Yanyuan Wu, Trinh Tran, Sami Dwabe, Marianna Sarkissyan, Juri Kim, Miguel Nava, Sheilah Clayton, Richard Pietras, Robin Farias-Eisner, Jaydutt V. Vadgama

**Affiliations:** 1 Division of Cancer Research and Training, Department of Medicine, Charles R. Drew University of Medicine and Science, 1731 East 120th Street, Los Angeles, CA 90059 USA; 2 David Geffen School of Medicine, Jonsson Comprehensive Cancer Center, University of California at Los Angeles, Los Angeles, CA USA

## Retraction Note: Breast Cancer Res Treat (2017) 163:449–460 10.1007/s10549-017-4211-y

The Editor-in-Chief has retracted this article. After publication, concerns were raised regarding image overlap with the authors' earlier article [1] and highly similar bands in the western blot images presented in the figures. Specifically:

- Figure 1d Twist, E-Cadherin and β-actin blots appear highly similar to the same proteins in Fig. 3a of [1];
- Figure 3d α-Tubulin blot appears highly similar to the same protein in Fig, 4c of [1];
- A number of bands in Figs. 1d, 2c, 2d, 3a, 3b, 3d, 4b, 4c, 5a, 5d and S2 appear to be duplicated.

Additionally, in Fig. 5b, the 4 h images for pSmad3 and pSmad3/DAPI don't seem to correspond to the same area, and two nuclei in the pSmad3/DAPI image appear highly similar.

The Editor-in-Chief therefore no longer has confidence in the presented data.

Yanyuan Wu and Jaydutt V. Vadgama do not agree to this retraction. Marianna Sarkissyan, Juri Kim, Miguel Nava, Sheilah Clayton, Richard Pietras and Robin Farias-Eisner have not responded to any correspondence from the editor or publisher about this retraction. The publisher has not been able to obtain current email addresses for Trinh Tran and Sami Dwabe.
